# London Students and Medical Degrees

**Published:** 1909-04-10

**Authors:** 


### London Students and Medical Degrees.

The long-standing problem touching the inaccessibility of medical degrees to students of medicine in London seems to be at last on the way to solution. This at least is the hopeful reflection which arises from perusal of the report recently issued upon the subject by the delegates of the Royal Colleges who were appointed last autumn to draft a scheme for establishing a system of joint examinations between the Colleges and the University of London. The draft has been made and adopted by both Colleges, and communicated to the University with a request that representatives of the latter should meet the College delegates and discuss the business. So the movement is well under weigh.

It is fully time the problem were grappled with, for several reasons. In the first place it is a decided hardship that the diplomate of the English Royal Colleges should lack the imprimatur of a medical degree although the tests to which he has successfully submitted himself are certainly not less severe than many of those which, out of London, lead directly to the coveted distinction. Moreover, this hardship is an important element in the reduction of numbers in the London medical schools. It would seem, therefore, that, from the standpoints both of equity and expediency, the time has fully arrived at which an attempt should be made to render medical degrees more attainable to London students than they have been in the past. The University and the Colleges draw to a very large extent upon an identical *clientèle*, and their fortunes are closely interwoven. Yet, under the present arrangement, a student desirous both of a London University degree and of a conjoint diploma must submit himself to two examinations in identical subjects—often within a few months. For the control of medical education in London, whether concerned with the University or with the Colleges, is largely in the same hands, while the general character of the examinations and the methods of conducting them are very similar in the two cases.

The need for greater co-ordination and simplicity in this matter of examination is emphasised in the report of the delegates, and it cannot be denied; for the existing practice is absurdly complicated, troublesome, and expensive. The points of contact between the University and the Colleges both in matters of *personnel* and of *matériel* are so numerous that the step to a more useful intimacy is not a long one, and seems, indeed, predestined. The delegates of the Colleges, at all events, have expressed a clear opinion that, provided there is a general desire on the part of the University, the Colleges, and the Medical Schools to unite in a concerted effort to provide an accessible degree in medicine, there should be little difficulty in arriving at an equitable and advantageous solution of the problem. They make the following suggestions:—That the University should retain all its existing rights as to the granting of degrees, but should consent to exercise them, *as regards Pass Degrees*, conjointly with the Royal Colleges so far as those students are concerned who shall have complied with such conditions as the University and the Royal Colleges may determine. By these means the University would continue to grant independent degrees in medicine and surgery, which would be of the nature of, and might be designated as, Honours Degrees, thus meeting the views of those who maintain that the present degrees of the University of London are of an Honours standard. The Royal Colleges, for their part, it is urged, should retain all their existing rights, and would continue independently to grant diplomas to those who are not eligible for the conjoint degree under the conditions laid down, or who do not desire it. The final recommendation is that the Colleges and the University should be associated for the conduct of all stages of the examination for the Pass Degrees (M.B., B.S., M.D.). It is maintained that the adoption of these suggestions will bring a Doctor's degree in Medicine within the reach of the average London student; that it will systematise medical education, and diminish the number of examinations which at present lie in the way of a man desirous both of a degree and a diploma; that by this means the natural advantages of London as a school of medicine will present themselves more clearly to students; and finally that a large amount of money will be saved.

